# Impaired cardiorespiratory and neuromuscular fitness in children and adolescents with juvenile idiopathic arthritis: a cross-sectional case–control study in the era of biologic drug therapies

**DOI:** 10.1186/s12969-023-00808-9

**Published:** 2023-03-17

**Authors:** Kati Räsänen, Kati Markula-Patjas, Saija Kantanen, Kalle Sipilä, Timo A. Lakka, Pekka Arikoski, Eija Piippo-Savolainen

**Affiliations:** 1 Pediatric Research Unit, University of Eastern Finland and Kuopio University Hospital, Kuopio, PL100, 70029 KYS Finland; 2 Center for Child Health Research, Tampere University and Department of Pediatrics, Tampere University Hospital, Tampere, Finland; 3 Department of Clinical Physiology and Nuclear Medicine, Faculty of Medicine and Health Technology, Tampere University and Tampere University Hospital, Tampere, Finland; 4 Institute of Biomedicine, School of Medicine, University of Eastern Finland, Kuopio Campus, Finland; 5 Department of Clinical Physiology and Nuclear Medicine, Kuopio University Hospital, Kuopio, Finland; 6 Foundation for Research in Health Exercise and Nutrition, Kuopio Research Institute of Exercise Medicine, Kuopio, Finland

**Keywords:** Juvenile idiopathic arthritis, Disease activity, Physical activity, Cardiorespiratory fitness, Neuromuscular fitness

## Abstract

**Background:**

In recent years, biologic drug therapies have altered the course of juvenile idiopathic arthritis (JIA) possibly also improving the patients’ physical fitness. However, studies measuring both cardiorespiratory and muscular fitness in children with JIA are sparse and have failed to show consistent results.

Our aim was to assess both cardiorespiratory and neuromuscular fitness and contributing factors in children and adolescents with JIA in the era of biologic drug therapies.

**Methods:**

This cross-sectional study consisted of 73 JIA patients (25 boys, 48 girls) aged 6.8- 17.5 years and 73 healthy age- and sex-matched controls, investigated in 2017–2019. Cardiorespiratory fitness was assessed by maximal ergospirometry and neuromuscular fitness by speed, agility, balance, and muscle strength tests.

**Results:**

Means (± SD) of maximal workload (W_max/kg_) and peak oxygen uptake (VO2_peak/kg,_) were lower in JIA patients than in controls (W_max/kg_: 2.80 ± 0.54 vs. 3.14 ± 0.50 Watts, *p* < 0.01; VO2_peak/kg_: 38.7 ± 7.53 vs. 45.8 ± 6.59 ml/min/kg, *p* < 0.01). Shuttle-run, sit-up and standing long jump test results were lower in JIA patients than in controls (*p* < 0.01). Mean (± SD) daily activity was lower (89.0 ± 44.7 vs. 112.7 ± 62.1 min/day, *p* < 0.05), and sedentary time was higher (427 ± 213 vs. 343 ± 211 min/day, *p* < 0.05) in JIA patients compared to controls. Physical activity and cardiorespiratory or neuromuscular fitness were not associated with disease activity.

**Conclusions:**

JIA patients were physically less active and had lower cardiorespiratory and neuromuscular fitness than their same aged controls with no JIA. Therefore, JIA patients should be encouraged to engage in physical activities as a part of their multidisciplinary treatment protocols to prevent adverse health risks of low physical activity and fitness.

## Background

Juvenile idiopathic arthritis (JIA) is one of the most common immune-mediated inflammatory diseases diagnosed in children younger than 16 years of age with the prevalence of 32.6 in 100,000 in Europe [1]. The symptoms of JIA include pain, joint stiffness, joint swelling, fatigue, and occasionally decreased physical function [2]. In recent years, developments in treatment modalities, including biologic drug therapies, have improved the overall outcome and physical performance in children with JIA [3]. However, pain-related fear of movement may inhibit physical performance and intensity of physical activity [4].

Studies measuring both cardiorespiratory and muscular fitness in children with JIA are sparse and the results have been inconsistent. Previous studies have demonstrated that children with chronic diseases have reduced physical activity (PA) despite disease activity [5, 6]. Low levels of physical activity in children and adolescents with JIA have been related to low anaerobic and aerobic fitness [7–9]. In a meta-analysis before the era of biologic drugs, maximal oxygen uptake, a measure of cardiorespiratory fitness (CRF), was 22% lower in JIA patients than in healthy children [10]. Also, other studies have reported lowered cardiorespiratory and neuromuscular fitness in children with JIA despite adequate treatment [11–14]. Van Brussel et al. observed that children with JIA had reduced anaerobic capacity [9]. A recent Canadian study in 2022 showed no statistically significant decrease in maximal oxygen uptake in a group of adolescents with JIA compared to same-aged controls [14]. Neither a larger Norwegian study in 2019 found a difference in CRF, assessed by a treadmill exercise test, between JIA patients and healthy children [13]. The explanation for this finding may have been that biologic drug therapies and multidisciplinary management had improved cardiorespiratory performance among JIA patients. As expected, however, muscle strength was lower in JIA patients compared to healthy children [13].

The golden standard method of evaluating CRF is a direct measurement of peak oxygen uptake by respiratory gas analysis during a maximal cardiopulmonary exercise test with a treadmill or a cycle ergometer [15]. Previous cohort and intervention studies have measured CRF by a maximal exercise test with or without respiratory gas analysis or by a submaximal exercise test without respiratory gas analysis [10, 13, 16–20]. In the submaximal test without respiratory gas analysis, the measurement of CRF is less precise than in the maximal exercise test with the direct measurement of peak oxygen uptake (VO2_peak_) by respiratory gas analysis. A commonly used submaximal test without respiratory gas analysis in children is the 6-min walk test [21]. In JIA patients, the 6-min walk test has been used to measure walking ability, but it has shown to be a poor measure of VO2_peak_ [19].

Good CRF is important for general health and has been found to be associated with a decreased preclinical atherosclerosis in children and a reduced risk of cardiovascular diseases in adults [22]. Differences in study designs, patient cohorts, disease activity, treatment modalities, as well as cardiorespiratory and neuromuscular fitness tests have led to inconsistent results on physical fitness in children and adolescents with JIA. The aim of our study was to assess cardiorespiratory and neuromuscular fitness in Finnish, caucasian children and adolescents with JIA and compare the results with healthy age- and sex-matched controls with most comprehensive and high-quality methods. Factors contributing to physical fitness in JIA patients were also investigated widely.

## Methods

### Study design

Cross-sectional case-controlled cohort study.

### Participants

Participants with JIA were recruited in two pediatric outpatient clinics in Kuopio and Tampere University Hospitals between 2017 and 2019. The first inclusion criteria was ongoing juvenile idiopathic arthritis diagnosed before the age of 16 years. The second inclusion criteria was the age of 6- 17 years enabling the performance of all physical tests. Exclusion criteria included physical or neurological disability, acute infection, or a long QT- syndrome preventing the performance of the physical tests.

The JIA patients had been diagnosed according to the criteria of International League of Associations for Rheumatology [23]. In this study, patients were divided into two disease groups by the number of affected joints ever: oligoarticular (less than five joints) and polyarticular (five or more joints) groups [23].

For each JIA patient, an age- and sex-matched control was selected from a Physical Activity and Nutrition in Children (PANIC) study over the period of 2007- 2017 (ClinicalTrials.gov: NCT01803776) [24]. The PANIC study is an ongoing population- based physical activity and dietary intervention study of 736 children who started the first grade in 16 primary schools in Kuopio in 2007–2009. A total of 70% of children (*n* = 512) were initially accepted to the baseline examination. They did not differ in sex distribution, age or body mass index- standard deviation score (BMI-SDS) from the children who started the first grade in 2007- 2009 in Kuopio. The comparison was based on data from the standard school health examinations performed for all Finnish children before the first grade. The PANIC study allocated the children to a combined physical activity and dietary intervention group (306 children, 60%) and to a control group (198 children, 40%). To avoid contamination by any health promotion programs, peers to our study were selected from the control group [20, 24, 25]. As there were no participants aged 11–14 years in the PANIC study, 15 healthy age- and sex-matched controls for JIA patients of this age were randomly selected through the National Registry of Finland and by a public recruitment procedure.

### Assessment of cardiorespiratory fitness

CRF in the Kuopio University Hospital and the Tampere University Hospital was assessed with a maximal exercise test using an electromagnetic cycle ergometer and a pediatric saddle module (Ergoselect 200 K, Ergoline, Bitz, Germany). Heart rate was measured by a 12-lead electrocardiography (Cardiosoft GE Healthcare Medical Systems, Version 6.5, Freiburg, Germany).

The study protocol included a 3-min warm-up period with a workload of 5 Watts (W). A 1-min steady-state period with a workload of 20 W was established followed by an exercise period with an increasing workload by 1 W per 6 s until voluntary exhaustion [20, 26]. In Tampere, the steady-state period was not recorded, and the initial load of 20 W increased constantly with 1 W every 6 s. The children were asked to keep the cadency stable within 70–80 rounds per minute, with a minimum of 65 rounds per minute. They were verbally encouraged to exercise until voluntary exhaustion. The exercise test was terminated and evaluated maximal if a child showed maximal effort and maximal cardiorespiratory capacity. The cycle ergometer test was regarded maximal if the heart rate was above 85% of the calculated age- standardized maximal heart rate [27]. Maximal absolute workload (W_max_) and maximal workload divided by body weight (W_max/kg_) were reported.

Respiratory gases were measured directly by the breath–by–breath method using a pediatric mask (in Kuopio Hans–Rudolph, Shawnee, Kansas, USA; in Tampere CPX Vyntus® or Oxycon Pro®, Jaeger, Hoechberg, Germany) from the beginning of the 2.5-min period sitting on the cycle before the exercise test until the post–exercise rest. VO2_peak_ was defined as the highest 15-s average value recorded during the last minute of the exercise test. VO2_peak/kg_ was also reported.

### Assessment of neuromuscular fitness

Running speed and agility were assessed by a 50-m shuttle run test where the child was asked to run a 5-m distance between two lines as fast as possible until 10 rounds were completed [28].

Abdominal and hip-flexor muscle strength and endurance were assessed with a sit-up test calculating the maximum number of sit-ups in 30 s [28]. Hand grip strength was measured by a vigorimeter (Martin, Tuttlingen, Germany) with three consecutive presses by both right and left hand. The best result of both hands was used in the analyses [29].

Lower limb explosive strength was assessed by a standing long jump test. The test score was the longest jump of three attempts in centimeters [30]. Static balance was assessed by a modified flamingo balance test [31, 32]. The children were asked to stand on one self-chosen leg with eyes closed for 30 s. The test score was the number of floor touches with a free foot or eye openings in 30 s.

Manual dexterity and upper limb movement speed were assessed by a box and block test [33]. The children were asked to remove small wooden cubes (2.5 cm per side) one by one with the dominant hand from one side of a wooden box (53.7 cm × 25.4 cm × 8.0 cm) to another side of the box in 60 s. They repeated the test with the non-dominant hand. The test score was the total number of removed cubes by each hand separately.

### Assessment of physical activity

Physical activity (minutes per day) and sedentary behavior including five weekdays and two weekend days (minutes per day) were assessed by the PANIC Physical Activity Questionnaire filled in by the parents together with their child at home [34]. The questionnaire had been validated earlier in a subsample of children participating in the PANIC study [34]. The types of physical activity included supervised physical activity, unsupervised physical activity, walking or cycling to and from school, and physical activity during holidays. The types of sedentary behavior included time spend on electronic media, music playing or listening, reading, writing, drawing and resting.

According to 2020 WHO guidelines, optimal physical activity time associated with improved health outcomes cannot be determined precisely. However, many of the benefits are observed with an average of 60 min of moderate-to-vigorous intensity physical activity daily [35].

The 48 JIA patients from the Kuopio University Hospital underwent cardiorespiratory and neuromuscular fitness tests using the protocols and facilities of the PANIC study [20, 26]. The 25 JIA patients from the Tampere University Hospital were tested in the Laboratory of Clinical Physiology of the hospital using the PANIC protocols.

### Disease activity

Disease activity was estimated by the Juvenile Arthritis Disease Activity score in 10 joints (JADAS-10, score range 0–40) that is a continuous disease activity score specific to JIA and consists of active joint count, physician’s global assessment of disease activity, parent’s or child’s evaluation of the overall well-being and erythrocyte sedimentation rate [36]. JADAS-10 cut off-values were used to divide the JIA patients into active disease (JADAS-10 ≥ 0.6 in oligoarthritis and JADAS-10 ≥ 0.8 in polyarthritis) and inactive disease (JADAS-10 < 0.6 in oligoarthritis and JADAS-10 < 0.8 in polyarthritis) [37].

Functional disability was assessed using the Childhood Health Assessment Questionnaire (C‐HAQ) scored 0–3 (0 = best and 3 = worst), pain using a 10‐cm visual analog scale (VAS) scored 0–10 (0 = no pain and 10 = worst pain) and global health using a 10-cm VAS scored 0–10 (0 = best and 10 = poorest) filled out by children or parents. Global disease activity was assessed by the physicians using a VAS (0 = no activity and 10 = maximum activity) [38].

### Statistical methods

The statistical analyses were performed with the IBM SPSS Statistics software, version 27 (IBM Corp., Armonk, NY, USA). The normality of the distributions of the variables was tested visually and by the Kolmogorov- Smirnov and Shapiro–Wilk tests. The independent sample t test, the Mann–Whitney U test or the Chi-square test were used to compare the JIA patients and the controls as well as the disease groups. Values were presented as means (standard deviations, SDs) for normally distributed variables and medians (interquartile ranges, IQRs) for variables with skewed distributions. Univariate linear regression analyses were used to assess possible correlates of measures of CRF and neuromuscular fitness. Variables that were statistically significantly associated with measures of CRF and neuromuscular fitness variables, were then entered into multivariate analyses. Results of regression analyses were reported as unstandardized coefficient B and as standardized beta values. All differences and associations with a *p*-value less than 0.05 were considered statistically significant.

## Results

### Patient demographics

This study cohort consisted of 73 patients (25 boys, 48 girls) with JIA, aged 6.8- 17.5 years, who were treated in the Department of Pediatrics of the Kuopio University Hospital (*n* = 48) or the Tampere University Hospital (*n* = 25), Finland, between years 2017 and 2019. Basic characteristics of patients and controls are shown in Table 1. In Kuopio, 54% of the eligible patients (*n* = 89) participated in the study, the corresponding data of the number of eligible patients from Tampere were not available. The JIA diagnoses included oligoarthritis (*n* = 18), extended oligoarthritis (*n* = 10), rheumatoid factor (RF)- negative polyarthritis (*n* = 41), psoriatic arthritis (*n* = 1), systemic arthritis (*n* = 1) and undifferentiated arthritis (*n* = 2). In our study group, there were no patients diagnosed with RF- positive polyarthritis or enthesitis-related arthritis. There was no difference in age, sex, body size, functional disability, or the prevalence of an active disease between JIA patients with a polyarticular disease and those with an oligoarticular disease. Age and- gender specific body mass index (ISO-BMI) was calculated with Finnish growth chart data (Table 1) [39]. The cumulative total glucocorticoid dose (intra-articular and systemic) in the past 24 months and disease-modifying anti-rheumatic drugs (DMARDs) and biologic DMARDs were more commonly used in patients of the polyarticular disease group. The number of patients with active disease did not differ statistically significantly between the two groups.

**Table 1:** Characteristics of patients with JIA and healthy controls (mean ± SD, unless indicated otherwise)

	**JIA**	**CONTROLS**	**P**	**OLIGO-** **ARTICULAR** **JIA**	**POLY-** **ARTICULAR** **JIA**	**P**
	*n* = 73	*n* = 73		*n* = 18	*n* = 55	
**Participant characteristics**						
Age, years	11.8 ± 3.01	11.7 ± 3.15	0.913	11.0 ± 2.94	12.0 ± 3.02	0.200
Gender,						
Male	25 (35%)	25 (35%)	1.00	5 (28%)	20 (36%)	0.508
Female	48 (65%)	48 (65%)		13 (72%)	35 (64%)	
Height, m	1.48 ± 0.16	1.50 ± 0.17	0.360	1.45 ± 0.17	1.49 ± 0.16	0.415
Weight, kg	43.8 ± 14.7	43.6 ± 14.6	0.941	40.5 ± 13.6	44.8 ± 15.1	0.287
ISO-BMI, kg/m^2^	23.4 ± 3.48	22.3 ± 3.11	0.058	22.3 ± 2.88	23.2 ± 3.14	0.803
**Disease characteristics**						
Disease duration, years	5.58 ± 3.32	NA	NA	4.88 ± 2.92	5.81 ± 3.44	0.308
Active disease	34 (55%)	NA	NA	7 (39%)	27 (49%)	0.458
C-HAQ score, median, IQR	0 (0–0.19)	NA	NA	0 (0–0.25)	0 (0–0.13)	0.928
NRS pain, median, IQR	0 (0–0.19)	NA	NA	2 (0–23.00)	3 (0–30.00)	0.754
NRS PGA, median, IQR	0 (0–1.50)	NA	NA	0 (0–0.50)	0 (0–3.00)	0.486
**Medication**						
Cumulative total ^a^glucocorticoid dose 12 months, mg, median, IQR	21.0 (0–160.50)	NA	NA	0 (0–65.00)	44.0 (0–265.25)	0.081
Cumulative total glucocorticoid dose 24 months, mg, median, IQR	116 (4.00–355)	NA	NA	40.0 (0–118)	172 (8.00–420)	0.032
Off medication	16 (22%)	NA	NA	8 (44%)	8 (15%)	0.008
DMARDs	57 (78%)	NA	NA	10 (56%)	47 (86%)	0.018
Biologic DMARDs	36 (49%)	NA	NA	5 (28%)	31 (56%)	0.026

*ISO-BMI* Age and gender-spesific body mass index, *JADAS-10* Juvenile idiopathic arthritis Disease Activity Score, *C-HAQ* Childhood Health Assessment Questionnaire, *NRS* Numerical rating scale, *PGA* Physician Global assessment of disease activity score, *DMARDs* Disease modifying antirheumatic drugs, *NA* Not applicable

^a^intra-articular and systemic

None of the JIA patients had severe disease-related or other symptoms such as pain or swelling of the joints which might have limited the exercise test performance. One patient had anemia, and none of the patients was a regular smoker. Twenty-three (32%) patients had ongoing physiotherapy. None of the patients had ongoing medication for asthma or other chronic lung diseases.

### Cardiorespiratory and neuromuscular fitness

Maximal workload (W_max/kg,_ Watts) and peak oxygen uptake (VO2_peak/kg_, ml/min/kg) divided by body weight were lower (mean ± SD) in JIA patients than in controls (Table 2). There were no differences in W_max/kg_ or VO2_peak/kg_ between JIA patients with a polyarticular disease and those with an oligoarticular disease.

**Table 2:** Physical fitness in patients with JIA and in healthy controls (mean ± SD)

	**JIA**		**Controls**		**Oligoarticular** **JIA**		**Polyarticular** **JIA**	
	*n* = 73		*n* = 73		*n* = 18		*n* = 55	
**Ergospirometry**^**a**^		n		n		n		n
W_max,_ Watts	119 ± 39.8	69	139 ± 50.9	72	113 ± 39.2	17	122 ± 40.2	52
W_max/kg,_ Watts/kg	2.80 ± 0.54**	69	3.14 ± 0.50	72	2.80 ± 0.52	17	2.80 ± 0.55	52
VO2_peak_, l/min	1.66 ± 0.57**	66	2.18 ± 0.58	53	1.50 ± 0.55	16	1.71 ± 0.57	50
VO2_peak/kg_, ml/min/kg	38.7 ± 7.53**	66	45.8 ± 6.59	53	36.4 ± 6.92	16	39.4 ± 7.64	50
**Neuromuscular**^**b**^** tests**								
Shuttle run, s	24.4 ± 3.58**	72	21.6 ± 1.93	68	24.5 ± 2.07	18	24.4 ± 3.97	54
Sit-ups, number/30 s	14.6 ± 5.55**	72	18.1 ± 5.61	70	12.5 ± 5.68	18	15.3 ± 5.39	54
Handgrip strength, kPa								
Right	66.3 ± 24.8	73	75.3 ± 28.0		66.7 ± 6.4 62.3		66.2 ± 24.5	
Left	64.3 ± 24.7*		74.1 ± 8.60	73	± 2.46	18	64.9 ± 25.6	55
Standing long jump, m	1.42 ± 9.52**	71	1.60 ± 3.90	68	1.40 ± 25.5	17	1.43 ± 30.9	54
Box and block, number/min								
Right	65.3 ± 11.9	71	65.1 ± 11.00		63.7 ± 9.45		65.9 ± 12.6	
Left	63.8 ± 11.2		63.6 ± 11.2	73	61.3 ± 8.28	18	64.6 ± 12.0	53
Balance test, support/30 s	2.01 ± 1.97	69	1.85 ± 1.73	52	2.22 ± 1.93	18	1.94 ± 2.00	51
**Physical activity min/day**								
Active time	89.0 ± 44.7*	62	112.7 ± 62.1	72	81.9 ± 46.4	16	91.5 ± 44.3	46
Sedentary time	427 ± 213*	65	343 ± 211	70	408 ± 173	16	432 ± 225	49

*W*_*max*_ maximal workload, *VO2*_*peak*_ peak oxygen uptake

^*^ *p* < 0.05, **p < 0.01 vs. controls

^a^Four patients could not keep the cadence stable because of not knowing how to cycle or did not find motivation for cycling. The missing respiratory gas measurements were due to unwillingness to use the mask

^b^ Due to lack of motivation, all tests were not completed by every patient

The 50 m shuttle-run test time was longer, the number of sit-ups in 30 s was smaller and standing long jump was shorter in JIA patients compared with controls. Flamingo balance test results did not differ between patients and healthy peers (Table 2).

None of the measures of CRF or neuromuscular fitness differed between JIA patients with an active disease and those with an inactive disease (Table 3). However, JIA patients in both of these groups had lower W_max/kg_ and VO2_peak/kg_, shorter 50-m shuttle-run test time, a larger number of sit-ups in 30 s, a longer standing long jump and a larger strength in the handgrip test.

**Table 3:** Physical fitness in JIA in relation to disease activity^a^ (mean ± SD) and healthy controls

	**Inactive disease**^**a**^		**Active disease**^**a**^		**Controls**	
	*n* = 39 (53%)		*n* = 34 (47%)		*n* = 73	
**Participant characteristics**						
Age, years	11.9 ± 3.18		11.6 ± 2.84		11.7 ± 3.15	
Gender						
Male	13 (33%)		12 (35%)		25 (34%)	
Female	26 (67%)		22 (65%)		48 (66%)	
**Ergospirometry**		n		n		n
W_max,,_ Watts	124 ± 36.6	37	115 ± 43.4*	32	139 ± 50.9	72
W_max/kg,_ Watts/kg	2.87 ± 0.56*	37	2.72 ± 0.51*	32	3.14 ± 0.50	72
VO2_peak_, l/min	1.70 ± 0.53*	34	1.61 ± 0.61*	32	2.18 ± 0.56	52
VO2_peak/kg_, ml/min/kg	39.3 ± 7.56*	34	38.0 ± 7.56*	32	45.8 ± 6.59	52
**Neuromuscular tests**						
Shuttle run, s	23.9 ± 2.73*	39	25.1 ± 4.34*	33	21.6 ± 1.93	67
Sit-ups, number/30 s	15.1 ± 5.68*	39	14.0 ± 5.42*	33	18.1 ± 5.61	70
Handgrip strength, kPa						
Right	66.1 ± 13.4	39	64.4 ± 9.78	32	75.3 ± 28.0	73
Left,	63.7 ± 12.5*		63.8 ± 9.71*		74.1 ± 28.6	
Standing long jump, m	1.41 ± 0.28*	39	1.43 ± 0.32*	32	1.60 ± 0.34	68
Box and block, number/min						
Right	67.0 ± 27.0	39	65.5 ± 22.4	34	75.3 ± 28.0	73
Left,	64.4 ± 27.3*		64.2 ± 21.8*		74.1 ± 28.6	
Balance test support/30 s	2.08 ± 1.99	39	1.93 ± 1.98	30	1.85 ± 1.73	52
**Physical activity min/day**						
Active time	89.2 ± 48.9	32	88.8 ± 40.5	30	112 ± 62.6	71
Sedentary time	423 ± 198	34	431 ± 231	31	343 ± 211	70

*W*_*max*_ Maximal workload, *VO2*_*peak*_ Peak oxygen uptake

^a^ JADAS-10 (Juvenile idiopathic arthritis Disease Activity Score cut off- values): -inactive disease JADAS-10 < 0.6 in oligoarthritis and JADAS-10 < 0.8 in polyarthritis; -active disease JADAS-10 ≥ 0.6 in oligoarthritis and JADAS-10 ≥ 0.8 in polyarthritis

^*^p < 0.05 vs. controls

JIA patients were physically less active and had higher sedentary time than controls (Table 2). There were no differences in physical activity or sedentary time between JIA patients with an active disease and those with an inactive disease or between oligo- and polyarticular disease groups (Table 3). A total of 43 (59%) JIA patients and 60 (82%) controls met the recommended 60 min of physical activity per day (*p* = 0.037) [35, 40]

### Correlates of cardiorespiratory and neuromuscular fitness in patients

Male JIA patients had higher W_max/kg_ (*P* = 0.003) and VO2_peak/kg_ (*P* = 0.001) than female JIA patients. In univariate analyses, higher sedentary time (β = 0.271, *p* = 0.038) and a higher ISO-BMI (β = -0.546, *p* < 0.001) were associated with lower W_max/kg_. Higher physical activity (β = 0.363, *p* = 0.006) and a lower ISO-BMI (β = -0.529, *p* < 0.001) were also associated with higher VO2_peak/kg_. For neuromuscular parameters, older age (β = -0.264, *p* = 0.025), female sex (β = 0.238, *p* = 0.044) and ISO-BMI (β = 0.337, *p* = 0.004) were correlates for shuttle run, where older age predicted better result, whereas female sex and higher ISO-BMI predicted inferior result.

Disease group, disease activity, biologic drug medication, antirheumatic drug therapy and cumulative total glucocorticoid treatment (intra-articular and systemic) at 12 or 24 months prior to study did not correlate with cardiorespiratory or neuromuscular parameters (data not shown).

In multivariate regression models, male sex and a lower ISO-BMI were independently associated with higher W_max/kg_, and male sex, higher physical activity and a lower ISO-BMI with higher VO2_peak/kg_. Older age was independently associated with shorter shuttle-run test time, older age and male sex with a longer standing long jump distance, and older age and a lower ISO-BMI with a larger number of sit-ups in 30 s (Table 4).

**Table 4:** Correlates of measures of cardiorespiratory and neuromuscular fitness in JIA patients in multiple regression analyses

Outcome	Unstandardized B	95% CI	Standardized β	*P*-value
**Wmax/kg (R**^**2**^ ** = 0.431)**				
Age	0.017	(-0.029, 0.066)	0.085	0.459
Female sex	-0.423	(-0.693, -0.153)	-0.341	0.003
Physical activity	0.003	(0.00, 0.008)	0.214	0.070
ISO-BMI	-0.121	(-0.162, -0.079)	-0.619	< 0.001
**VO**_**2**_ **peak/kg (R** ^**2**^ ** = 0.374)**				
Age	0.209	(-0.413, 0.830)	0.084	0.503
Female sex	-6.565	(-10.051, -3.079)	-0.439	< 0.001
Physical activity	0.045	(0.005, 0.085)	0.285	0.028
ISO-BMI	-1.138	(-1.721, -0.555)	-0.447	< 0.001
**50-m shuttle run (R** ^**2**^ ** = 0.200)**				
Age	-0.383	(-0.645, -0.122)	-0.395	0.005
Female sex	1.431	(-0.80, 2.942)	0.244	0.063
Physical activity	-0.004	(-0.021, 0.014)	-0.057	0.680
ISO-BMI	0.212	(-0.022,0.446)	0.230	0.075
**Long jump (R** ^**2**^ ** = 0.473)**				
Age	5.09	(3.186,6,997)	0.586	< 0.001
Female sex	-19.5	(-30.527, -8.463)	-0.372	0.001
Physical activity	0.023	(-0.105, 0.150)	0.040	0.722
**Sit-up (R** ^**2**^ ** = 0.311)**				
Age	1.043	(0.585,1.501)	0.569	< 0.001
Female sex	-0.697	(-3.345, 1.951)	-0.063	0.600
Physical activity	0.021	(-0.010, 0.051)	0.175	0.176
ISO-BMI	-0.413	(-0.823, -0.003)	-0.237	0.049

*JIA* juvenile idiopathic arthritis, *W*_*max*_ maximal workload, *VO2*_*peak*_ peak oxygen uptake, *ISO-BMI* Age and gender-spesific body mass index

## Discussion

We observed that CRF, measured as W_max/kg_ and VO2_peak/kg_ by ergospirometry, was lower in children with JIA than in controls, and VO2_peak/kg_ correlated positively with higher daily physically active time. No differences were found between patients with active or inactive disease or between those with oligoarticular or polyarticular disease.

An earlier meta-analysis, performed prior to the era of biologic drug therapies in 2002, reported impaired CRF, measured as maximal oxygen uptake (VO2_peak_), in children with JIA compared to controls [10]. The measurements in the meta-analysis had been performed by cycle ergometer in four studies, which is comparable to our methodology, and with treadmill in one study. The numbers of JIA patients and controls in these studies were relatively small, but the ages of the JIA patients were similar to those of ours. However, the findings in the individual studies were not consistent, and in one study, CRF did not differ significantly between the patients and the controls, probably due to selection bias. Two of the studies suggested that more severely affected JIA patients were less physically fit.

Contrary to the findings of our study and the conclusions of the meta-analysis [10], a more recent Norwegian study observed no difference in VO_2peak_, measured by a treadmill exercise test, between JIA patients and controls [13]. Our patients were physically less active and had higher sedentary time than controls, whereas no such differences were found in the Norwegian study [41]. The disease activity did not differ between our and the Norwegian JIA patients. The treatment protocols were also similar since DMARDs and biologic DMARDs were administered comparably. Therefore, the lower physical activity and the higher sedentary time among JIA patients compared with controls in our study but not in the Norwegian study could explain the difference in physical performance between JIA patients in these studies. Our finding thus emphasizes the importance of regular physical activity and avoidance of sedentary behavior in maintaining adequate CRF among JIA patients. It has been shown that kinesiophobia may hinder physical performance and functional quality of life in JIA [4]. In our study, no correlation was observed between pain and decreased active time. Further research is needed to elucidate the significance of kinesiophobia in JIA and to show whether pain is the contributing factor lowering physical activity [4]. A recent study assessing the impact of psychosocial stress factors on physical activity, observed that decreased physical activity was associated with higher disease activity and higher disease-specific psychosocial stress [42]. Taking psychosocial factors into consideration is important because they can have multiple effects on children with JIA, including physical activity in daily life. These aspects will be investigated in our future research.

A recent case–control study by Nesbitt et al. in 2022 reported a tendency towards lower CRF in 29 JIA patients and lower levels of physical activity in 26 JIA patients compared to typically developing peers [14]. However, they concluded that a larger number of patients is needed to determine whether the observations would reach statistical significance. In our larger study, CRF was significantly lower, and decreased VO2_peak/kg_ in 66 measured JIA patients was directly associated with low physical activity.

We observed that male sex was associated with higher W_max/kg_ and VO2_peak/kg_ in JIA patients. This could be explained by a larger increase in maximal stroke volume, muscle mass, and blood hemoglobin concentration during puberty in boys than in girls [43]. In the Norwegian study, male patients had higher VO2_peak_, and also Nesbitt et al. observed higher peak oxygen consumption in males [13, 14].

As in the Norwegian study, also we found that a lower body mass index was associated with higher VO2_peak/kg_ in JIA patients [13]. A recent study by Pepera et al. 2022 observed that healthy children with a normal BMI had better CRF than obese and overweight children [44]. In 2010, Goran et al. reported that the major influence of body weight on VO2_peak_ is explained by fat free mass and that excess fatness has a detrimental effect on submaximal aerobic capacity [45].

Muscle strength measured by long-jump and sit-ups tests was lower, and agility, assessed by shuttle run test, was poorer in our JIA patients than in controls which is in line with previous studies [12, 13, 46]. However, direct comparisons are difficult to make due to varying measurement methods and different muscle groups involved. In a smaller study, muscle architecture and force did not differ between JIA and healthy children, but poorer functional abilities were observed in vertical jump performance. The investigators speculated that JIA patients may show pain avoiding behavior during multiarticular dynamic activities [47, 48]. Kunzte et al. also reported that in JIA patients there are multijoint movement alterations in vertical drop jump which may be a factor leading to the difference in muscle strength and performance between JIA and healthy children [49].

When evaluating static balance, Flamingo test results in our patients did not differ from those of controls. Houghton et al. [50] observed a correlation between poor balance and lower extremity weakness. Low disease activity and lack of articular limitations in our patients may have resulted in beneficial effects on their proprioception.

As the course of JIA fluctuates, including remission and active phases, the results of CRF and neuromuscular fitness tests could be affected by their timing. Therefore, restrictions due to acute JIA phase could impair daily physical activity. However, there is some previous evidence that the activity of JIA does not correlate with the level of physical activity [6]. In line with this observation, we found no association between disease activity and CRF or neuromuscular fitness. Neither daily active time nor sedentary time differed between those with active disease or inactive disease in our study. However, there is evidence that JIA impacts biomechanical features of gait and the motion of hip, knee and ankle joints. These changes may be alleviated by exercise or pharmaceutical interventions [51].

One of the strengths of our study was the age- and sex-matched control group from the population-based ongoing PANIC-study cohort, representing a general population of Finnish school-aged children and adolescents. To complete the control group, we additionally randomly selected healthy children through the National Registry and by a public recruitment procedure. We used the same measurement protocols for patients and controls, and the tests were performed in highly experienced laboratories by well-trained personnel and with modern facilities. In Finland, the number of school children meeting the recommendations for adequate physical activity has increased during the 2000s [40]. Even though the participants for the PANIC study were examined between 2007 and 2017, our findings can be considered reliable since the difference in CRF and neuromuscular measurements between our patients and controls could have been even larger with a more recent control cohort. Data on the characteristics of participants were carefully collected, and disease characteristics for each patient were evaluated by a pediatric rheumatologist.

A possible limitation of our study was that 54% of the JIA patients treated in the outpatient clinic of the Kuopio University Hospital agreed to participate. The corresponding data of the number of eligible patients from Tampere were not available. This could have led to the bias of more physically fit patients being recruited. However, we were able to show the impairment of physical performance even in our potentially selected JIA patient group emphasizing the reliability of our findings. Another limitation might have been that no power analysis was conducted due to multiple variables assessed in our study. The predominance of polyarthritis (75%) over oligoarthritis (25%) in our study cohort was comparable to the distribution of JIA-diagnoses in our area.

## Conclusions

In the era of biologic drugs, we found that JIA patients had lower CRF and neuromuscular fitness, were physically less active and had more sedentary time compared with age- and sex matched healthy controls. We also observed that CRF, muscle strength and agility were directly associated with physical activity in JIA patients.However, larger multicenter studies are warranted for further evaluation of factors that can affect JIA patients’ CRF, such as disease activity and type, physical activity and psychosocial stress. Good CRF in children and adolescents has been found to be associated with less advanced preclinical atherosclerosis. JIA patients should be encouraged to engage in physical activity as a part of their multidisciplinary treatment protocols to prevent adverse health risks of low physical activity and fitness.

## Data Availability

The data used and /or analysed during the study are only available from the corresponding author on reasonable request.

## Abbreviations

BMI: Body mass index
CHAQ: Child Health Assessment Questionnaire
CRF: Cardiorespiratory fitness
DMARD: Disease modifying drug
ILAR: International League of Associations for Rheumatology
ISO-BMI: Age and gender-specific body mass index
JADAS: Juvenile Arthritis Disease Activity Score
JIA: Juvenile idiopathic arthritis
PA: Physical activity
RF: Rheumatoid factor
SD: Standard deviation
VAS: Visual analogic scale
VO2_peak_: Peak oxygen uptake
VO2_peakk/kg_: Peak Oxygen uptake per kilogram
WHO: World Health Organization
W_max_: Maximal workload
W_max/kg_: Maximal workload per kilogram
